# Targeting Dermatologic Side Effects of Immunotherapy Using Novel Skin Care Products

**DOI:** 10.7150/jca.126265

**Published:** 2026-01-01

**Authors:** Marithé Claes, Joy Lodewijckx, Jolien Robijns, Laura Tuts, Melissa Lenaerts, Eline Vandaele, Tim Wessels, Annelies Requilé, Daisy Luyten, Yolanda Verheezen, Eric Joosens, Jeroen Mebis

**Affiliations:** 1 Hasselt University, Faculty of Medicine and Life Sciences, Agoralaan, 3590 Diepenbeek, Belgium / LCRC.; 2 Jessa Hospital, Dept. Medical Oncology and Dept. Jessa & Science, Salvatorstraat 20, 3500 Hasselt, Belgium / LCRC.

**Keywords:** cancer, immunotherapy, emollient, oncology, skin care, quality of life, skin toxicity

## Abstract

**Objective:** Immunotherapy can be accompanied by cutaneous adverse events that negatively impact the patient's quality of life (QoL). This trial aimed to evaluate the efficacy of two novel skin care products in preventing and managing cutaneous adverse events associated with immunotherapy.

**Methods:** An interventional, open-label, single-group, pretest-posttest study was conducted at the Jessa Hospital (Belgium) involving cancer patients receiving immunotherapy (n=75). Patients applied the skin care products daily for three weeks. A researcher evaluated skin toxicity using the National Cancer Institute - Common Terminology Criteria for Adverse Events (NCI-CTCAE) v5.0. Questionnaires assessed the frequency and severity of their symptoms (Numeric Rating Scale, NRS), the patient's QoL (Dermatology Life Quality Index, DLQI, and Skindex-29), the Patient Benefit Index, and patient satisfaction (NRS).

**Results:** The CTCAE and NRS showed that pruritus and xerosis were the most frequently observed skin toxicities. According to the NCI-CTCAE, an improvement was detected in the grade of pruritus and xerosis after applying the novel emollients (P<0.001). All patient-reported symptoms decreased significantly in frequency. Both the Skindex-29 total score (P<0.001) and DLQI (P=0.038) improved over time. Moreover, 42.7% of the patients experienced at least one patient-relevant benefit of the treatment. Lastly, 70.7% of the patients were satisfied with the products, and 74.6% would recommend them to other patients.

**Conclusion:** This trial demonstrates that the two novel emollients effectively alleviate immunotherapy-related dermatological toxicities. As a result, an improvement in the patient's QoL was observed, accompanied by high satisfaction and a strong likelihood of recommendation. Future research with a control group is necessary to draw firm conclusions.

## Introduction

Over the course of this century, cancer may surpass cardiovascular diseases as the leading cause of death worldwide.^1^ Nevertheless, significant advancements have been made in cancer treatment. Historically, this treatment has centred around surgery, radiotherapy, and chemotherapy, but the increasing success of immunotherapy has rendered it a crucial factor in cancer therapy.^2^ Immune checkpoint inhibitors are an important type of immunotherapy. They target immune checkpoints, receptors on the surface of immune cells that regulate the activation or suppression of immunological responses. These checkpoints regulate the innate reactions of the immune system to tumour growth, as immune system activation is necessary for tumour control but can also lead to autoimmunity.^3^, ^4^ They can overcome tumour-induced immunosuppression by inhibiting these checkpoints and facilitating immune-mediated tumour clearance.^4^

Unfortunately, immunotherapy can be accompanied by adverse events that negatively impact the patient's quality of life (QoL). These immune-related side effects manifest as inflammatory reactions against the host's healthy tissue and are often characterised by cutaneous symptoms.^3^, ^5^ An estimated 30 to 50% of patients receiving immunotherapy will experience dermatological toxicities.^2^, ^6^ These cutaneous adverse effects most commonly manifest as pruritus, nonspecific maculopapular rash, eczema, lichenoid eruptions, and psoriasiform rash.^2^, ^5^, ^6^ In extreme cases, these dermatological toxicities can be dose-limiting and affect the efficacy of the treatment.^2^, ^5^

Management of cutaneous immune-related adverse events is a stepwise process and may rely on the use of corticosteroids.^2^, ^3^, ^5^, ^6^ Glucocorticosteroids, however, may affect the anti-tumour response and are linked to several possible adverse effects.^6^ The management of pruritus , a common cutaneous adverse event, is based on topical solutions (e.g., moisturisers, camphor/menthol, or steroids) and non-sedating antihistamines.^5^, ^6^ Specific guidelines on the management of cancer treatment-related dermatological toxicities were developed by the leading organisations in the field of oncology and supportive cancer care, such as The American Society of Clinical Oncology (ASCO), the Multinational Association of Supportive Care in Cancer (MASCC), National Comprehensive Cancer Network (NCCN), and the European Society of Medical Oncology (ESMO).^7^-^10^ Nonetheless, for some interventions, the evidence of recommendation is moderate to insufficient. Therefore, it is essential to explore other potential management strategies for dermatological complications of immunotherapy.^11^, ^12^

For this trial, a hydrating, hypoallergenic, anti-itching body serum and face cream were developed to tackle cutaneous adverse events of immunotherapy. This trial aims to evaluate the effectiveness of these two novel skin care products in preventing and managing cutaneous adverse events associated with immunotherapy.

## Methods

### Study design and setting

This trial evaluated the effectiveness of a body serum and face cream for immunotherapy-associated cutaneous toxicities using a prospective, monocentric, interventional, open-label, single-group, pretest-posttest study. This clinical trial was conducted at the Jessa Hospital (Hasselt, Belgium) between October 2021 and August 2023. Ethical approval was obtained by the ethics committees of the Jessa Hospital and Hasselt University (B2432021000015). This study was conducted in accordance with the Declaration of Helsinki and was registered at ClinicalTrials.gov (NCT04929834).

### Study population

All adult cancer patients undergoing immunotherapy (checkpoint inhibitors) at the Jessa Hospital (Hasselt, Belgium) were eligible for this trial. Patients were excluded when they had pre-existing skin conditions unrelated to their cancer treatment (e.g., psoriasis or eczema) or if they received concomitant chemotherapy or radiotherapy. Moreover, patients could not enter the trial when they had any psychological disorder or unstable medical condition that could affect the safety of the patient and their compliance in the study as judged by the investigator. Written informed consent was obtained before the start of the trial.

### Intervention

Participating patients received two novel skin care products: a hydrating, anti-itching facial emollient and a body lotion, which they applied daily for three weeks. The emollient contains camphor, sodium ascorbyl phosphate, xylitylglucoside, anhydroxylitol, xylitol, sodium hydroxide, caprylic/capric triglyceride, methyl lactate, tocopherol, dimethicone, shea butter oleyl esters, carbomer, glycerine, caprylyl glycol, and aqua. The body lotion consists of camphor, menthyl lactate, urea, sodium lactate, lactic acid, triacetin, caprylyl glycol, caprylic/capric triglyceride, sodium ascorbyl phosphate, dimethicone, cetyl alcohol, tocopherol, hydrogenated ethylhexyl olivate, hydrogenated olive oil unsaponifiable, hydroxyethyl acrylate, sodium acryloyldimethyl taurate copolymer, polyacrylate cross polymer-6, t-butyl alcohol, xanthan gum, glycerin, C14-22 alcohols, C12-20 alkyl glucoside, and aqua. During this three-week study period, the patients were advised not to use other skin care products besides sunscreen when appropriate.

### Primary outcome measures

#### Skin Reaction Evaluation

The National Cancer Institute - Common Terminology Criteria for Adverse Events version 5 (NCI-CTCAE v5.0) were used to evaluate the immunotherapy-related cutaneous toxicities.^13^ The criteria were applied by a trained researcher at baseline and at the end of the three-week study period to assess the following skin reactions: xerosis, pruritus, erythroderma, erythema multiforme, acne, bullous dermatosis, maculopapular rash, induration, skin atrophy, ulceration, Stevens-Johnson syndrome, and epidermal necrolysis.

#### Patient benefit

Patients scored their treatment needs and benefits using the Patient Benefit Index (PBI), a standardised questionnaire comprising two components. The first component, the Patient Needs Questionnaire, was administered at baseline and evaluates the importance of various treatment objectives from the patient's perspective. The second component, the Patient Benefit Questionnaire, was collected at the end of the study and assessed the patient's perspective on the success of the treatment. A global score can be calculated by weighting the benefit values according to importance. The data are scored from 0 to 5 (0-4 corresponds to 'not at all' to 'very much'; 5 stands for 'does/did not apply to me'). Each benefit item is multiplied by the respective importance item, after which the product is divided by the sum of all importance items. The resulting global score can range from 0 (no benefit) to 4 (highest possible benefit).^14^

### Secondary outcome measures

#### Symptom evaluation

Patients scored the intensity and frequency of five common immunotherapy-related skin reactions (dryness, pruritus, burning sensation, pain, and erythema) on an 11-point Numeric Rating Scale (NRS, 0 = no symptom, 10 = worst symptom) using an online questionnaire. These symptoms were evaluated at baseline, weekly, and after three weeks.

#### Quality of life

The patient's QoL was measured using two validated questionnaires for QoL correlated to skin conditions: the Skindex-29 and the Dermatology Life Quality Index (DLQI). The Skindex-29 consists of three scales evaluating the functioning (12 items), emotions (10 items), and symptoms (7 items). Each item is scored on a five-point scale (0 = never, 100 = all the time), with a higher total score representing a poorer QoL. A total score of ≥ 25 implies a mild impact of the skin reactions on the QoL, while a score of 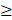
32 indicates a moderate effect and a score 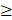
44 suggests a severe impact.^15^, ^16^ The DLQI questionnaire comprises 10 items scored on a four-point Likert scale (0 = not at all, 3 = very much), with a higher total score illustrating a more significant impairment of the QoL. Their skin condition severely impacts the patient's QoL if the total score exceeds 10.^17^ These questionnaires were administered at baseline and the end of the study period.

#### Patient satisfaction

Patients scored their overall satisfaction regarding the pleasantness and soothing effect of the skin care products, as well as their willingness to recommend the products to other patients, using a 5-point NRS (not at all satisfied/willing to recommend, not satisfied/recommend, neutral, satisfied/recommend, extremely satisfied/highly recommend). The satisfaction score was collected at the end of the study.

### Other outcomes of interest

#### Patient data

General patient-, disease-, and treatment-related information was obtained through a questionnaire and the patient's medical records.

### Statistical analysis

The normality of the data was checked using the Shapiro-Wilk test. Categorical values are displayed as numbers and the corresponding percentages, while continuous data are shown as the median, 25^th^, and 75^th^ percentiles. The Wilcoxon Signed-Rank and Friedman tests, as appropriate, were used to analyse the continuous data. Bonferroni correction was applied to counteract multiple post-hoc testing. The statistical significance level for all analyses was set at 5% (p < 0.05, two-tailed). All analyses were performed using SPSS 28.0 (IBM, Chicago, IL).

## Results

### Patient characteristics

**Figure 1:**
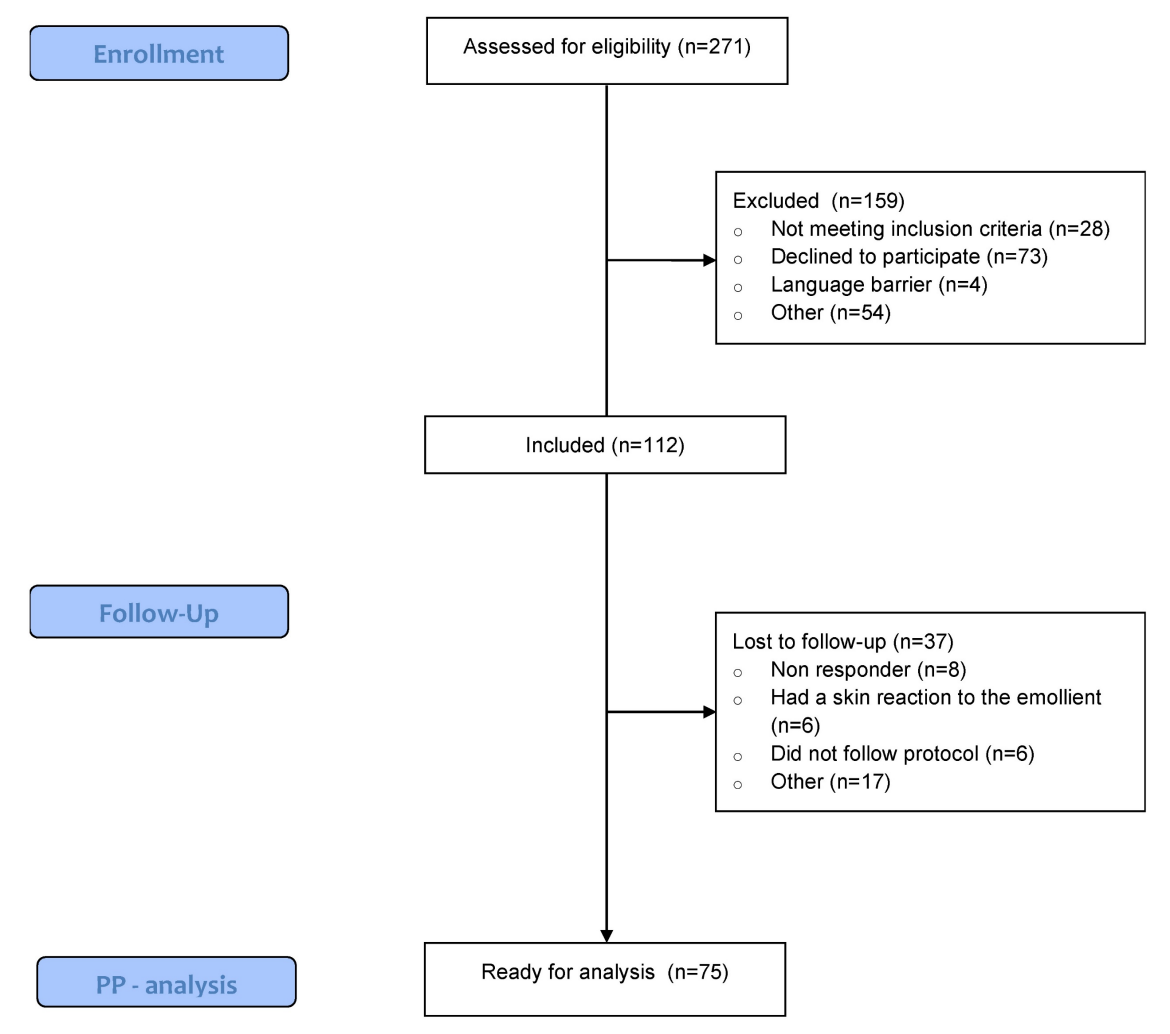
** Consort flow chart.** PP: Per protocol.

Between October 2021 and August 2023, 271 patients undergoing immunotherapy were screened for eligibility. Of those, 41% met the inclusion criteria and were willing to participate. Ultimately, 37 patients were lost to follow-up due to not adhering to the protocol, experiencing a skin reaction to the emollient, or failing to respond to the questionnaires. A total of 75 patients were included in the per-protocol analysis (Figure 1). Table 1 displays the patient-, disease-, and treatment-related characteristics of the patients. The study population was predominantly female (64% vs. 36% males), with a mean age of 63.17. The most common primary tumour location was skin (36%), followed by breast (17.3%). Pembrolizumab (54.7%) and Nivolumab (30.7%) were the most frequently administered immunotherapy types, and patients started their immunotherapy a median of 20.85 weeks prior to trial enrollment.

### Skin toxicity

#### NCI-CTCAE v5.0 criteria

**Figure 2:**
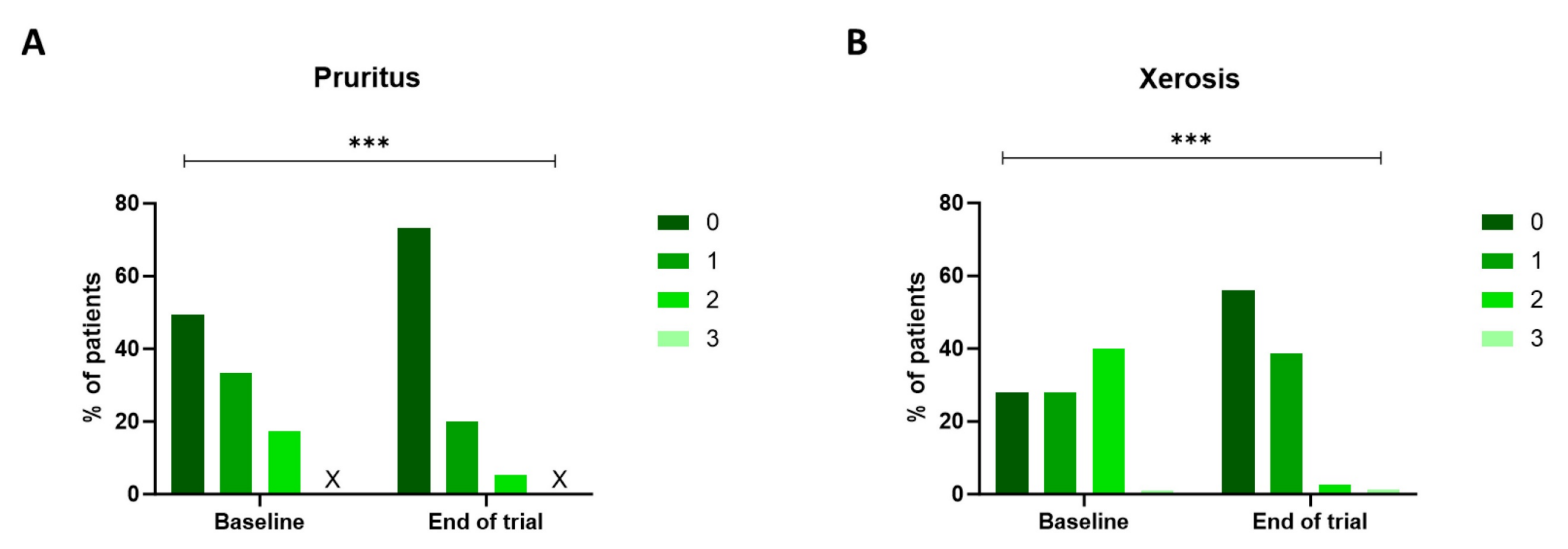
** NCI-CTCAE v5.0 grading for pruritus and xerosis.** The degree of pruritus (A) and xerosis (B) was assessed using the NCI-CTCAE v5.0 scale at baseline and at the end of the trial. The severity of both pruritus and xerosis decreased during the three-week trial (p<0.001, Wilcoxon Signed-rank test). The 'X' indicates that no patients fit this category. NCI-CTCAE v5.0: National Cancer Institute - Common Terminology Criteria for Adverse Events version 5.

Xerosis and pruritus were the most observed out of all twelve skin toxicities based on the NCI-CTCAE v5.0 criteria. At the same time, no cases of bullous dermatosis, acne, induration, Stevens-Johnson Syndrome, ulceration, or epidermal necrolysis were detected. The remaining types of cutaneous adverse events (erythroderma, erythema multiforme, maculopapular rash, and skin atrophy) were observed in a limited number of patients at baseline (respectively, 1.3%, 4%, 6.7%, and 1.3%). At the start of the trial, 50.6% of all patients experienced some degree of pruritus, with the majority classified as NCI-CTCAE grade 1. Grade 2 pruritus, indicating an effect on the patient's instrumental activities of daily living, was observed in 17.3%. After the study period, only 25.3% of patients experienced any degree of pruritus (P<0.001), of which 5.3% were classified as grade 2. Moreover, 72% of the patients suffered from xerosis. Of them, 28% were classified as grade 1, 40% as grade 2, and 4% as grade 3. These percentages decreased significantly post-treatment with the novel skin care products, as only 2.7% experienced a grade 2 xerosis at the end of the trial (P<0.001) (Figure 2).

#### NRS scale

**Figure 3:**
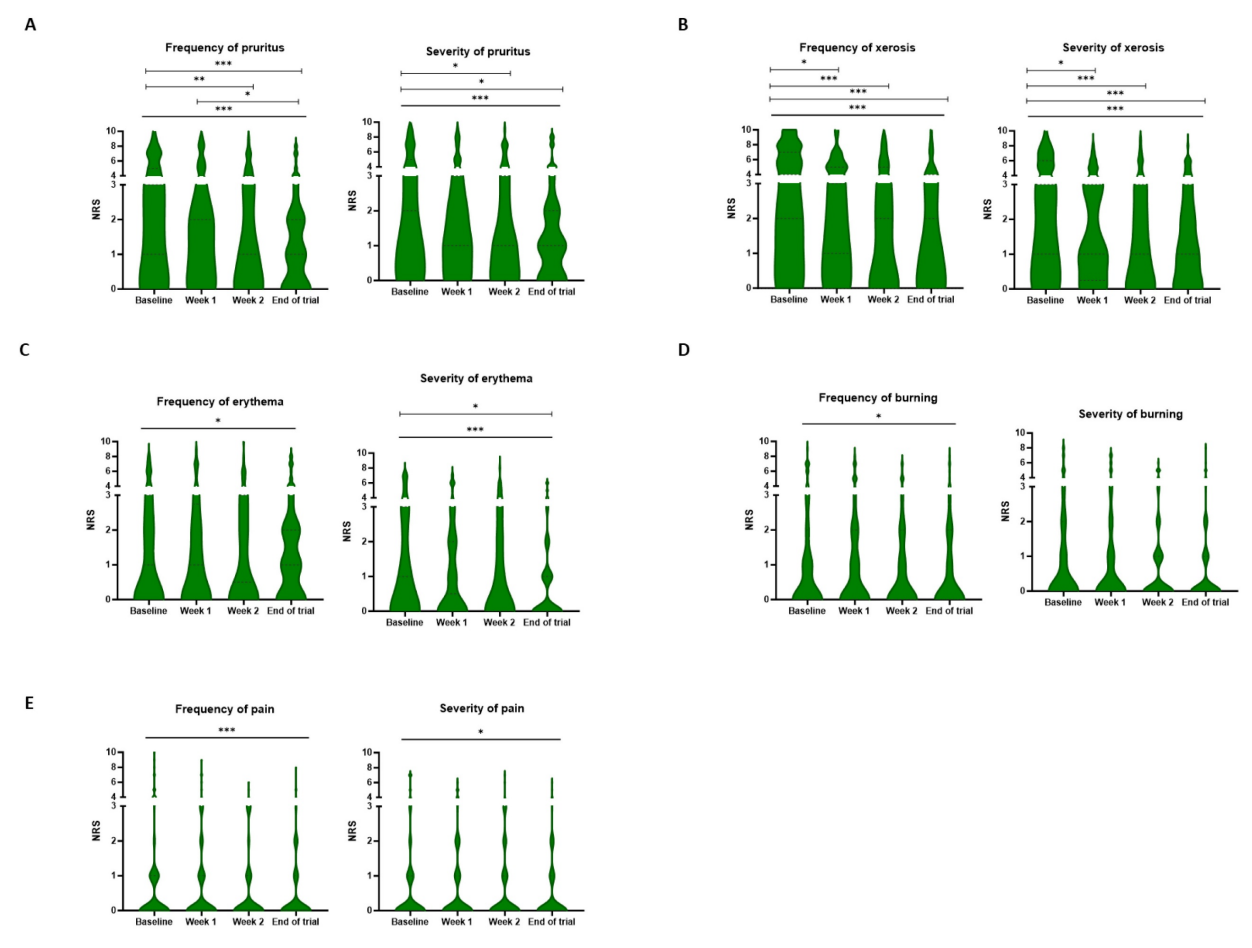
** Frequency and severity of patient-rated cutaneous symptoms.** The patients scored the frequency and severity of pruritus (A), xerosis (B), erythema (C), burning (D), and pain (E) on a numeric rating scale at baseline, weekly, and the end of the trial. A significant decrease was observed in the frequency of all symptoms over time. Only the severity of burning did not decrease significantly during the study period. * P < 0.05, ** P < 0.01, *** P < 0.001, Friedman analysis and post-hoc pairwise comparison with Bonferroni correction. NRS: Numeric rating scale.

A significant improvement in the frequency of all patient-related symptoms was observed after treatment with the novel skin care products (Ps<0.05). Moreover, the severity of all symptoms except for burning (P=0.054) decreased significantly over time. Again, xerosis and pruritus were the most common toxicities. For the frequency of xerosis, the NRS score decreased from a median score of 4 [2-7] at baseline to 2 [0-4] at the end of the trial (P<0.001). This improvement could also be observed in the severity of xerosis, as the score evolved from 3 [1-6] at baseline to 1 [0-3] at the three-week mark (P<0.001). The emollients alleviated the severity and frequency of pruritus as the score, respectively, decreased from 2 [0-5] and 3 [1-5] at baseline to 1 [0-2] and 1 [0-2] at the end of the trial (Figure 3).

### Quality of life

#### Skindex-29

**Figure 4:**
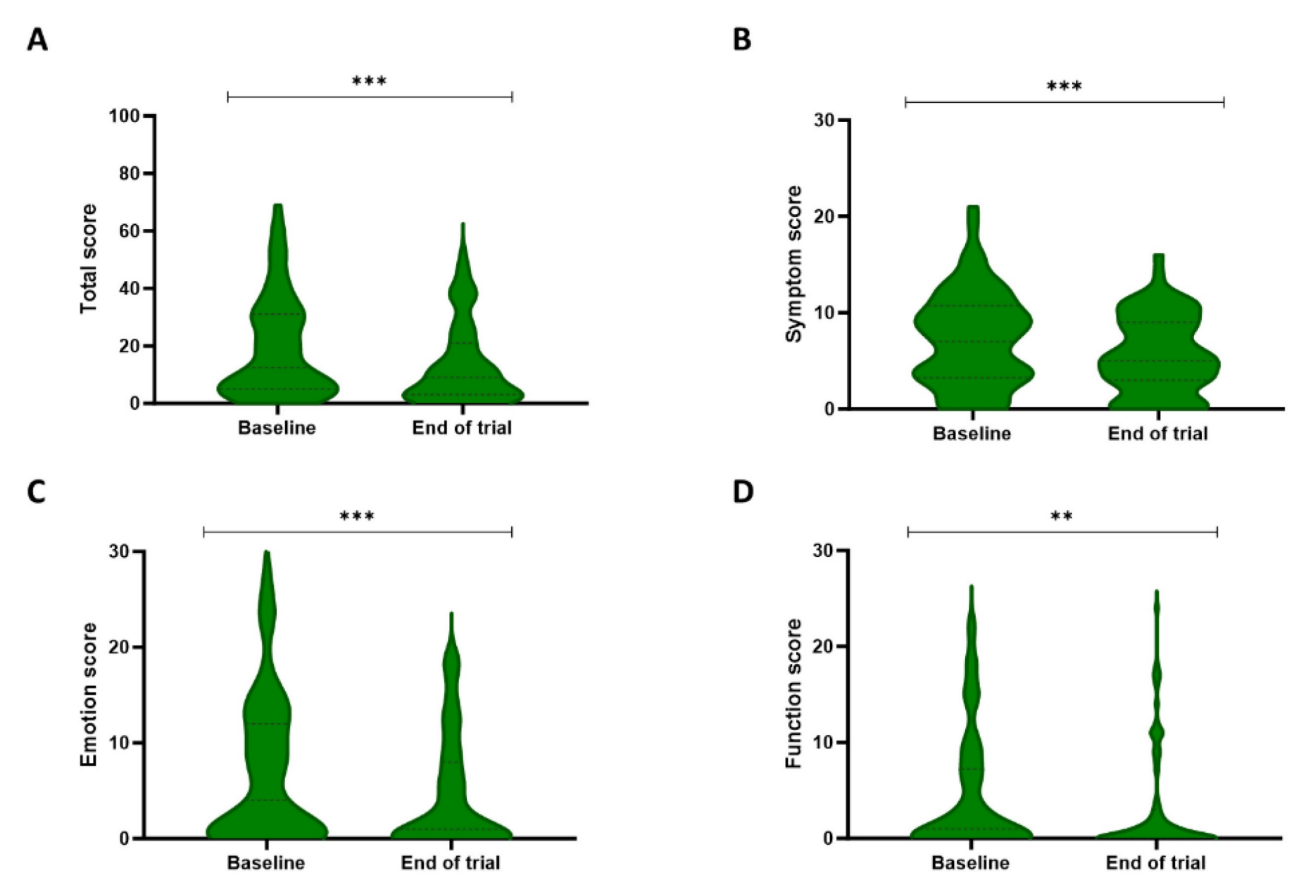
** Patient's quality of life evaluated by the Skindex-29 questionnaire.** The Skindex-29 questionnaire describes the quality of life of the patients on a total score (A), a symptom subscale (B), an emotion subscale (C), and a functioning subscale (D). The total score and all three subscales showed significant improvement between baseline and the end of the three-week study period. ** P<0.01, *** P<0.001, Wilcoxon Signed-rank test.

Analysis of the Skindex-29 total score and subscales (emotion, symptoms, and functioning) showed significant differences over time (Figure 4). More specifically, the total score decreased from a median of 12.5 [5-31] at baseline to 9 [3-21] post-treatment (P < 0.001). Additionally, the symptom score reduced from 7 [3.25-10.75] to 5 [3-9] during the trial (P < 0.001). Lastly, a decline could be detected in both the function and emotion sub-scores, with respective scores of 1 [0-7.25] and 4 [0-12] at baseline and 0 [0-3] and 1 [0-8] after the trial (respectively, P = 0.007 and P < 0.001).

#### DLQI

**Figure 5:**
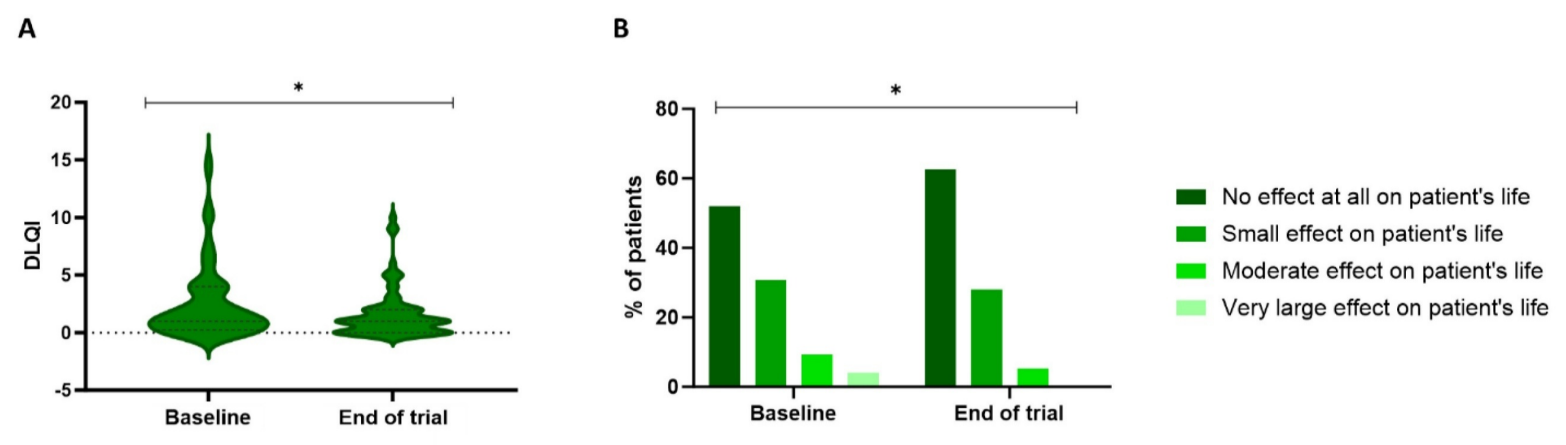
** Patient's quality of life based on the DLQI questionnaire.** The DLQI scores (A) can be used to classify the patients based on how severely their cutaneous toxicity affects their quality of life (B). A significant improvement was observed in the DLQI scores during the trial (P=0.038, Wilcoxon Signed-rank test). DLQI: Dermatology Life Quality Index.

*Figure 5* illustrates the significant difference in the DLQI over time (P=0.038). At baseline, 52% of the patients experienced no effect of skin toxicity on their QoL, 30.7% experienced a small effect, 9.3% a moderate effect, and 4% a substantial effect. After treatment with the skin care products, 62.7% experienced no impact on their QoL, 28% had a small effect, and 5.3% had a moderate effect. These effects were reflected in a DLQI score of 1 [0.25-4] at baseline and 1 [0-2] at the end of the trial.

### Patient benefit and satisfaction

The assessment of the patient benefit resulted in a median PBI score of 1 [0.32-1.86]. Furthermore, 37 patients had a PBI score equal to or higher than 1, which indicates that 49.3% of the patients experienced at least one patient-relevant benefit of the treatment. Lastly, analysis of the satisfaction and recommendation scores showed that 70.7% of the patients were satisfied with the products, and 74.6% would recommend them to other patients.

## Discussion

The results of this trial showed that xerosis and pruritus were the most commonly observed cutaneous toxicities related to immunotherapy. Furthermore, the novel skin care products effectively reduced the severity and frequency of the immunotherapy-related skin toxicities evaluated by the patient. Moreover, the researchers detected a significant improvement in xerosis and pruritus after a three-week trial period, reflected by an increase in the QoL andhigh satisfaction and recommendation grades.

Immunotherapy-related cutaneous toxicities remain a concern for cancer patients.^3^, ^5^ Therefore, this clinical trial focused on two novel skin care products and their effect on these dermatologic symptoms. The most prevalent immunotherapy types administered in this trial were Pembrolizumab and Nivolumab, both anti-programmed death receptor-1 (PD-1) monoclonal antibodies (mAbs). PD-1, a receptor primarily expressed on activated CD8+ T cells, is responsible for downregulating tumour-specific T-cell responses.^18^ Adverse effects of anti-PD-1 mAbs can occur in the endocrine system, lungs, liver, and gastrointestinal tract. However, the majority of anti-PD-1 side effects are dermatologic, but a severe manifestation is often rare.^19^ The researcher-reported and the patient-reported outcomes (PROs) showed that pruritus and xerosis were the most observed symptoms. *Ji H.H. et al. (2019*) concluded that pruritus and rash were the most common adverse events in patients receiving anti-PD-1 mAbs. The incidence of rash in patients treated with Pembrolizumab and Nivolumab was 13-21% and 15-22%, respectively.^19^ In comparison, only five patients (6.7%) experienced maculopapular rash in this trial. Moreover, *Wu J. et al. (2019)* disclosed an incidence of pruritus of 17-18.8% for Nivolumab and 14.1-20.7% for Pembrolizumab. This incidence increased to 33.2-47% when Nivolumab was combined with the anti-CTLA4 mAb Ipilimumab.^20^ In our trial, 50.6% of the included patients experienced some degree of pruritus, as assessed by the researcher at baseline. Moreover, when questioned at baseline, 50.67% gave an NRS score greater than three on the frequency of their itch, indicating mild to severe pruritus. In our trial, all patients undergoing immunotherapy were informed, but patients who experienced symptoms of dermatologic toxicities were often more inclined to participate. Therefore, an inclusion bias was created as this limited the number of patients who did not experience any symptoms.

Over the years, oncodermatology has evolved as studies have shown that including dermatologists in the cancer team decreases the recurrence of dermatological toxicity.^21^, ^22^ The MASCC and the Association Francophone des Soins Oncologiques de Support (AFSOS) reached an international expert consensus on the role of dermocosmetics in managing cancer-related skin toxicities and provided general recommendations.^21^ While dermatological care in cancer patients should be individualised, general daily skin care is recommended from the start of their treatment as it maintains and supports the skin microbiota and epidermal skin barrier. This general skin care should include cleansers, moisturisers, and photoprotection when appropriate. For pathological, sensitive, and fragile skin, it is recommended that the formulations be free of irritants (e.g., perfumes or fragrances), additives, herbal extracts, and sensitising agents.^21^, ^23^ To battle xerosis and the accompanying pruritus, emollients may include shea butter to improve the skin barrier and urea to hydrate and exfoliate.^21^ Both ingredients can be found in the skin care products tested in this trial.

A patient's QoL may be impacted by the psychological and physical side effects of dermatological toxicities brought on by anti-cancer treatment.^24^-^26^ Moreover, studies showed that appropriate skin care during cancer treatment improved the patient's QoL.^24^ At baseline, both questionnaires showed that most of the included patients reported a limited effect of their cutaneous symptoms on their QoL. However, we could still detect a significant improvement over time, possibly due to the decrease in pruritus experienced by the patients. Severe persistent pruritus significantly affects the physical and symptom components of QoL scores and may extend to the social and emotional domains.^26^, ^27^ Pruritus persistence can lead to irritability, which could affect the emotional parameters. By appropriately managing skin toxicity, we could improve the emotional parameters and, therefore, the QoL.^26^

Unfortunately, this clinical trial also had some limitations. Firstly, we did not conduct a randomised controlled trial. It was challenging to design this trial, including a control group, as no standard skin care products are generally approved. Moreover, the per-protocol design resulted in numerous drop-outs. Patients were excluded if they did not apply the emollient for the entire study period or failed to answer the online questionnaires. However, non-compliance could be due to their satisfaction with the study product, which could have affected the results. A last limitation is the diversity of our study population. The broad inclusion criteria resulted in heterogeneity in patient characteristics, including cancer type, stage, and type of immunotherapy. The strength of this study lies in the method of data collection. The results were obtained by combining PROs and researcher-reported outcome measures, providing a subjective perspective from the patient and a more objective perspective from the researcher. Additionally, all questionnaires and grading tools used in this trial were validated.

Future studies are necessary to optimise the non-pharmaceutical standard care for cutaneous toxicities following immunotherapy. These studies should be designed as a double-blinded, randomised controlled trial to increase power and minimise bias. Diversity could be limited by focusing on a single type of immunotherapy or primary cancer type, and follow-up should be extended to assess the long-term effects. Lastly, it could be intriguing to study the preventative action of the emollients by including patients before starting immunotherapy.

## Conclusion

This pretest-posttest trial in cancer patients undergoing immunotherapy shows that the two novel emollients alleviated immunotherapy-related dermatological toxicities. As a result, this improved the patient's QoL and resulted in high satisfaction and recommendation grades. Future research with a control group is necessary to draw firm conclusions.

## Figures and Tables

**Table 1:** Patient-, disease- and treatment-related characteristics.

Characteristic	Analysed patients (n=75)
	*Mean* 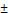 *SD*
Age (years)	63.17 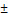 13.27
BMI (kg/m²)	26.61 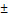 5.72
	*%*
Gender	
Female Male	64%36%
Smoker	
CurrentFormerNever	10.7% 34.7% 54.7%
Alcohol consumption	
< 1 per week 1-3 per week 3-10 per week 10-20 per week	58.7% 24% 4% 13.3%
WHO skin type classification ^a^	
Melano-compromised Melano-competent Melano-protected	21.3% 69.3% 9.3%
Tumour location	
Skin Breast Lung Kidney Colorectal Bladder Head and Neck Ureter Endometrium Brain Stomach Prostate Other	36% 17.3% 14.7% 10.7% 4% 2.7% 2.7% 2.7% 1.3% 1.3% 1.3% 1.3% 1.3%
T-stage	
X ^b^ In situ 1 2 3 4	17.3% 1.3% 13.3% 28% 21.3% 16%
N-stage	
X ^c^ 01 2 3	22.6% 36% 22.7% 6.7% 12%
M-stage	
X ^d^ 01	2.7% 56% 41.3%
Immunotherapy type ^e^	
Pembrolizumab Nivolumab Ipilimumab Durvalumab Atezolizumab Avelumab Dostarlimab Bevacizumab Other	54.7% 30.7% 5.3% 4% 2.7% 2.7% 1.3% 1.3% 2.7%
	*Median (P25-P75)*
Duration of immunotherapy (weeks)	20.86 (5.57-40.43)

BMI, Body Mass Index; SD, standard deviation; WHO, World Health Organization; P25: 25^th^ percentile; P75: 75^th^ percentile. ^a^ The WHO skin type classification is based on the Fitzpatrick's scale: melano-compromised (Fitzpatrick's skin type I- II), melano-competent (Fitzpatrick's skin type III-IV), and melano-protected (Fitzpatrick's skin type V-VI). ^b^ Main tumour cannot be measured ^c^ Cancer in nearby lymph nodes cannot be measured ^d^ Metastasis cannot be measured ^e^ The percentages will not add up as some patients receive a combination of different types of immunotherapy.

## Abbreviations

AFSOS: Association Francophone des Soins Oncologiques de Support
ASCO: American Society of Clinical Oncology
DLQI: Dermatology Life Quality Index
ESMO: European Society of Medical Oncology
LCRC: Limburg Clinical Research Centre
MASCC: Multinational Association of Supportive Care in Cancer
mAbs: Monoclonal Antibodies
NCI-CTCAE: National Cancer Institute - Common Terminology Criteria for Adverse Events (NCI-CTCAE)
NRS: Numeric Rating Scale
NCCN: National Comprehensive Cancer Network
PBI: Patient Benefit Index
QoL: Quality of Life
